# *In vivo* characterization of the role of tissue‐specific translation elongation factor 1A2 in protein synthesis reveals insights into muscle atrophy

**DOI:** 10.1111/febs.12554

**Published:** 2013-10-23

**Authors:** Jennifer Doig, Lowri A. Griffiths, David Peberdy, Permphan Dharmasaroja, Maria Vera, Faith J. C. Davies, Helen J. Newbery, David Brownstein, Catherine M. Abbott

**Affiliations:** ^1^ Medical Genetics SectionMolecular Medicine CentreInstitute of Genetics and Molecular MedicineWestern General HospitalUniversity of EdinburghUK; ^2^ Anatomy & Structural BiologyAlbert Einstein UniversityBronxNYUSA; ^3^ Research Animal Pathology Core FacilityQueen's Medical Research InstituteUniversity of EdinburghUK; ^4^ Department of AnatomyFaculty of ScienceMahidol UniversityRoom B128Bangkok10400Thailand

**Keywords:** motor neuron degeneration, muscle atrophy, transgenic mice, translation elongation

## Abstract

Translation elongation factor 1A2 (eEF1A2), uniquely among translation factors, is expressed specifically in neurons and muscle. eEF1A2‐null mutant wasted mice develop an aggressive, early‐onset form of neurodegeneration, but it is unknown whether the wasting results from denervation of the muscles, or whether the mice have a primary myopathy resulting from loss of translation activity in muscle. We set out to establish the relative contributions of loss of eEF1A2 in the different tissues to this postnatal lethal phenotype. We used tissue‐specific transgenesis to show that correction of eEF1A2 levels in muscle fails to ameliorate the overt phenotypic abnormalities or time of death of wasted mice. Molecular markers of muscle atrophy such as Fbxo32 were dramatically upregulated at the RNA level in wasted mice, both in the presence and in the absence of muscle‐specific expression of eEF1A2, but the degree of upregulation at the protein level was significantly lower in those wasted mice without transgene‐derived expression of eEF1A2 in muscle. This provides the first *in vivo* confirmation that eEF1A2 plays an important role in translation. In spite of the inability of the nontransgenic wasted mice to upregulate key atrogenes at the protein level in response to denervation to the same degree as their transgenic counterparts, there were no measurable differences between transgenic and nontransgenic wasted mice in terms of weight loss, grip strength, or muscle pathology. This suggests that a compromised ability fully to execute the atrogene pathway in denervated muscle does not affect the process of muscle atrophy in the short term.

**Abbreviations**
ALS: amyotrophic lateral sclerosis
eEF1A: translation elongation factor 1A
eEF1A1: translation elongation factor 1A1
eEF1A2: translation elongation factor 1A2
GAPDH: glyceraldehyde‐3‐phosphate dehydrogenase
HSA: human skeletal actin
MND: motor neuron disease
NSE: neuron‐specific enolase
SMA: spinal muscular atrophy
SMN: survival motor neuron protein

## Introduction

Motor neuron disease (MND) is the umbrella term for a group of neurodegenerative diseases primarily affecting the motor neurons of the spinal cord. The term covers both the early‐onset genetic disorder spinal muscular atrophy (SMA) and the adult‐onset forms of MND, both sporadic and genetic. The adult form is often referred to as amyotrophic lateral sclerosis (ALS). MND has a complex aetiology that has, until recently, often eluded characterization at a genetic level, with a few notable exceptions, many involving defects in genes encoding molecules involved in RNA processing 1, together with a recent discovery of a gene whose product is involved in protein degradation 2. Severe muscle atrophy is seen in MND, and the mechanisms underlying this process have been studied in many animal and cellular models.

A number of mouse models exist for both SMA and ALS forms of the disease. In some cases, these involve targeted mutations in genes that have previously been implicated in human forms of the disease, but there are also mouse mutants that model the neurodegenerative process without necessarily involving the same genetic basis. One such model is the wasted mouse, which arises from a spontaneous recessive mutation resulting in deletion of the promoter region and first noncoding exon of the *Eef1a2* gene encoding translation elongation factor 1A2 (eEF1A2). This 15.8‐kb deletion completely abolishes eEF1A2 protein expression 3. Mice homozygous for this deletion develop an early‐onset aggressive form of motor neuron degeneration, characterized by muscle atrophy, tremors and gait abnormalities soon after weaning. They then deteriorate rapidly and die, typically by 28 days 4. The onset of this phenotype coincides with a developmental switch in eEF1A variants in muscle.

There are two independently encoded translation elongation factor 1A (eEF1A) isoforms in mammals, translation elongation factor 1A1 (eEF1A1) and eEF1A2. Whereas eEF1A1 is almost ubiquitously expressed, its expression in muscle starts at high levels at birth, but then declines to become almost undetectable by ~ 21 days. Meanwhile, eEF1A2 levels in muscle rise from very low levels at birth to high levels by 21 days after birth 5. The loss of eEF1A1 in muscle thus corresponds precisely to the onset of the wasted phenotype. Within brain and spinal cord, there is exclusive expression of one or other eEF1A isoform, depending on cell type, with neurons expressing only eEF1A2 and glial cells expressing only eEF1A1 6. Wasted mice therefore have neither eEF1A1 nor eEF1A2 expression in muscle or motor neurons beyond 21 days of age. As eEF1A is essential for *de novo* protein synthesis, it is assumed that both muscle cells and neurons will be severely affected by the mutation. Indeed, in addition to the loss of muscle bulk, motor neuron pathology is found in wasted mice 3. From 17 days, nerves retract from motor endplates, and by 19 days reactive gliosis is seen in spinal cord sections. Neurofilament accumulation in the perikarya of motor neurons is then seen, and vacuolation and death of motor neurons is observable by 24 days.

Previous transgenic studies in our laboratory have shown that *Eef1a2* is the only gene responsible for the wasted phenotype 6. However, it has never been established whether the loss of muscle bulk, loss of muscle function and ultimate death of wasted mice results from the loss of eEF1A2 in muscle, or whether the muscle phenotype is caused by denervation atrophy resulting from loss of eEF1A2 in the motor neurons. This is an important question, as studies on other MND‐related genes have given contrasting results. Gavrilina *et al*. 8 showed in a mouse model of SMA that transgenic expression of the survival motor neuron protein (SMN) from a neuronal promoter completely ameliorated the symptoms and rescued the phenotype of the mice. However, expression of SMN exclusively in muscle [driven by the human skeletal actin (HSA) promoter] failed to rescue the phenotype, suggesting that the muscle atrophy seen in these mice is entirely neurogenic in origin. This was further confirmed by the authors' analysis of a line expressing SMN from the HSA promoter, in which leaky low‐level expression was seen in neurons. In this line, the mice survived for 160 days, showing that even a low level of expression in neurons has more impact on the phenotype than high‐level expression in muscle. In contrast, a similar study was carried out in a mouse model of SMA with respiratory distress, which is caused by mutations in the *Ighmbp2* gene. In this case, the phenotypic abnormalities could only be corrected by expression of the wild‐type gene in both muscle and neurons 9. These contrasting results suggest that there are different mechanisms underlying the neuromuscular problems caused by mutations in distinct genes.

eEF1A2 has not been directly implicated in human neurodegenerative disease, but a specific missense mutation in eEF1A2 has been found in two individuals with severe intellectual disability and epilepsy 11. A subunit of the GTP exchange factor for eEF1A, eEF1B2, has also been associated with severe intellectual disability 13. A copy number variant in another subunit of this complex, eEF1D, has been tentatively associated with ALS 14.

In all cases of muscle atrophy studied, whether as a result of starvation, disuse, cancer, or denervation, a coordinated programme of changes in gene expression is seen. These coordinately regulated genes have been termed ‘atrogenes’ (for atrophy‐related genes) 15. Among the most widely studied atrogenes are Fbxo32 (also called atrogin‐1 or MAFbx) and MuRF1, both of which are substantially upregulated in response to muscle damage. They encode muscle‐specific E3 ubiquitin ligases that are regarded as key molecules for the control of the process of atrophy, targeting specific proteins for proteasomal degradation 16. Although these atrogenes have been extensively assayed, there is still some controversy about whether they are actually essential for the process of muscle atrophy. Murton, for example, states ‘indeed, while unlikely, the assumed elevation of MAFbx and MuRF1 mRNA levels culminating in a decline in muscle mass may merely be the result of guilt by association’ 17. On the other hand, there is very good evidence for a functional role for these factors from knockout mouse studies, where loss of function of either gene led to a reduction in denervation‐induced muscle atrophy 18.

We set out to establish whether the muscle atrophy seen in the wasted mouse could be corrected or ameliorated by expression of eEF1A2 solely in muscle, or whether the muscle atrophy was primarily neurogenic in origin. This allowed us to assess the potential of the wasted mouse mutant as a model for the process of motor neuron degeneration; we showed that restoration of eEF1A2 in muscle but not in neurons fails to rescue the wasted phenotype. We also assayed molecular markers of muscle atrophy in wasted mice with and without muscle‐specific eEF1A2 transgenes. Both transgenic and nontransgenic wasted mice showed significant levels of upregulation of markers of muscle damage as assayed by quantitative RT‐PCR, but wasted mice without the transgene showed much more modest increases in these markers than transgenic wasted mice when they were assayed by western blotting. This finding is consistent with the predicted (but not hitherto directly demonstrated) role of eEF1A2 as a crucial translation factor. The availability of mice all undergoing denervation atrophy, but both with (*wst*/*wst* transgenic) and without (*wst*/*wst* nontransgenic) the ability to convert the increases in markers of muscle atrophy seen at the RNA level into similar increases at the protein level, provides a novel system for investigation of the mechanisms of muscle atrophy.

## Results

### Experimental design

In order to establish whether the weight decline, loss of grip strength and other abnormalities seen in wasted mice result from the loss of eEF1A2 activity in muscle and/or neurons, we set out to dissect out different aspects of the phenotype by using different promoters to drive tissue‐specific expression of eEF1A2 in transgenic mice. These were crossed with +/*wst* heterozygous mice, and then intercrossed to generate transgenic *wst*/*wst* homozygotes, which were characterized in terms of expression of eEF1A2 and by phenotypic assays.

The tissue‐specific promoters that we used were designed to force expression of eEF1A2 in neurons only [the rat neuron‐specific enolase promoter (NSE)] and in muscle only (the human skeletal actin promoter, HSA). A 1.8‐kb fragment of the promoter region of the gene encoding NSE has been previously shown to contain all of the regulatory elements required for driving neuron‐specific expression in transgenic mice 19, and numerous studies have demonstrated neuron‐specific transgene expression, using this promoter region 10. The HSA promoter has been extensively used in transgenic mice 21; it is expressed in all skeletal muscle types from birth onwards, reflecting the expression pattern of eEF1A2 in muscle. In each case, the promoter fragment was ligated to all coding exons (2–8) of human eEF1A2. We have previously shown that human eEF1A2 can functionally substitute for mouse eEF1A2 in transgenic mice 6, and the use of a human construct allowed us to design RT‐PCR assays to distinguish between expression of endogenous and transgene‐derived eEF1A2.

### NSE–eEF1A2

Pronuclear injection of the NSE–eEF1A2 construct into fertilized mouse oocytes gave rise to three transmitting founder mice. Only two of the resulting lines (called line B and line C) showed clear expression of the transgene in brain at the RNA level, and were thus chosen for further analysis.

**Figure 1:**
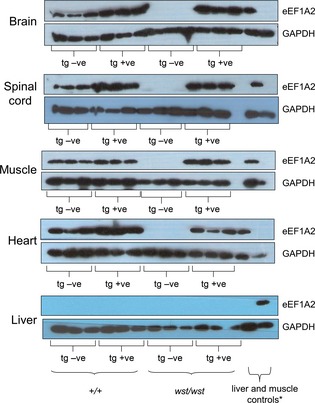
Western blots showing expression of eEF1A2 and GAPDH (as a loading control) in tissues from transgenic and nontransgenic mice of the transgenic line NSE–EEF1A2‐B. Expression from three mice from each group (wild type, wild type transgenic, wasted, and wasted transgenic) is shown. *The final two lanes contain muscle (as an eEF1A2‐positive control) and liver (as an eEF1A2‐negative control) samples; in the top four sections, the order is muscle and then liver on the extreme right, but in the bottom panel the order is reversed, and liver is loaded first, with muscle on the extreme right.

**Figure 2:**
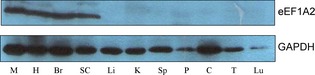
Western blots showing expression of eEF1A2 and GAPDH (as a loading control) on an expanded panel of tissues from a wasted mice carrying the transgene from the transgenic line NSE–EEF1A2‐B. M, muscle; H, heart; Br, brain; SC, spinal cord; Li, liver; K, kidney; Sp, spleen; P, pancreas; C, colon; Lu, lung.

With the exception of a single amino acid, human and mouse eEF1A2 are identical at the amino acid level. This meant that we were unable to examine protein expression of the transgene until the final stage of the crosses, when homozygosity for the wasted allele meant that there was no confounding expression of endogenous eEF1A2. When tissues from transgene‐positive homozygous wasted mice were subjected to western blot analysis, we found that both lines B and C unexpectedly had expression of the transgene in both brain and muscle; this could be attributable to position effects, but the promoter that we generated by PCR incorporated an artificial restriction site to facilitate cloning, and we cannot exclude the possibility that this sequence affected the specificity of expression. Line B was chosen for further analysis. Figure 1 shows western blots of eEF1A2 in various tissues from three mice of each genotype, all at 25 days: wild‐type mice with and without the transgene, and *wst*/*wst* homozygotes with and without the transgene. It is clear that, whereas (as expected) there was no eEF1A2 expression in any of the nontransgenic wasted mice, levels of eEF1A2 in muscle and heart of transgenic wasted mice were comparable to or even higher than those seen in nontransgenic wild‐type mice [average densitometry reading relative to glyceraldehyde‐3‐phosphate dehydrogenase (GAPDH) of 4.4 in wasted transgenic muscle as compared with 1.5 in wild‐type controls]. Similarly, expression of eEF1A2 in brain and spinal cord of all transgenic mice was higher than that seen in wild‐type mice (average densitometry reading relative to GAPDH of 6.7 in wasted transgenic muscle as compared with 2.1 in wild‐type controls for brain, and 1.4 as compared with 1.3 in spinal cord), so all transgenic *wst*/*wst* mice had levels of eEF1A2 that were at least as high as those of their nontransgenic littermates in brain, spinal cord, muscle, and heart. Increased expression of the transgene was more marked in brain and spinal cord than in muscle, consistent with the known properties of the NSE promoter. The bottom panel of Fig. 1 shows that there was no expression of either endogenous or transgene‐derived eEF1A2 in liver, as expected, and Fig. 2 shows eEF1A2 expression in an extended panel of tissues from a transgenic wasted mouse, showing that there was no apparent ectopic expression of the transgene other than in muscle.

Immunohistochemistry of eEF1A2 in spinal cord and brain in the transgenic wasted mice (i.e. where there is no confounding expression of the endogenous gene) showed expression of eEF1A2 exclusively in neuronal populations, and particularly strong expression in Purkinje cells in the cerebellum, and in the motor neurons of the spinal cord (Fig. S1), reflecting the normal pattern of expression of eEF1A2. In earlier work, we showed that, in addition to its high‐level expression in neurons and muscle, eEF1A2 is also expressed in specific cell types, such as a subset of pancreatic islet cells 6. We therefore also used immunohistochemistry to examine expression in pancreas, and found that, as expected for the NSE promoter, the transgene was not expressed in pancreatic islet cells (Fig. S1, right panel), in contrast to endogenous eEF1A2. There was no evidence of effects on the pancreas of these mice when sections were examined (data not shown).

Analysis of transgenic wasted mice as compared with their nontransgenic littermates revealed complete correction of the wasted phenotype. The mice appeared healthy and bred normally; most mice were culled along with normal littermates between 2 and 6 months, but a subset were retained with no apparent ill‐effects for 12 months, so we have no evidence that the lifespan was reduced relative to wild‐type mice. They had spleens and thymuses of normal size, and showed no tremors or gait abnormalities. Body weights at 25 days are given in Table 1, and show that, whereas nontransgenic *wst*/*wst* mice were significantly smaller than their wild‐type littermates, those that expressed the transgene achieved a similar weight to wild‐type mice, with a significant difference (*P* < 0.01) being seen between transgenic and nontransgenic homozygous wasted mice. The presence of the transgene in non‐wasted mice had no significant effect on body weight. The NSE–eEF1A2 transgene thus corrects the loss of muscle bulk seen in wasted mice, as the majority of body weight loss derives from a decrease in muscle weight 7. It is worth noting that the overexpression seen in muscles and neurons of wild‐type mice carrying the transgene appeared to cause no ill‐effects.

**Table 1:** Body weights of mice from NSE–eEF1A2 line B at 25 days. *P < 0.01 for comparison of wasted mice without the transgene with wild‐type mice; those with the transgene are not significantly different from wild‐type mice. SEM, standard error of the mean.

Genotype at wasted locus	Transgene +/−	Weight (g) ± SEM	No. of mice
+/+	−	12.7 ± 0.3	13
+/+	+	12.1 ± 0.5	13
+/*wst*	−	11.5 ± 0.3	15
+/*wst*	+	11.6 ± 0.4	9
*wst*/*wst*	−	7.3 ± 0.6*	8
*wst*/*wst*	+	11.7 ± 0.4	6

We then went on to test muscle function by analysing grip strength in the mice (Table 2). At 25 days of age, towards the end‐stage of disease, analysis of grip strength normalized to body weight in both forelimbs only and all four limbs together showed that wasted mice carrying the transgene (+/*tg*,* wst*/*wst*; ‘tg’ is used to designate the presence of the transgene in the gene symbol) were indistinguishable from wild‐type mice (+/+, +/+), and had significantly higher grip strength than wasted mice without the transgene (+/+, *wst*/*wst*). This difference was slightly more marked when only forelimbs were analysed. The poor muscle function seen in wasted mice is therefore completely corrected by expression of the transgene.

**Table 2:** Mean grip strength analysis of 25‐day‐old mice from NSE–eEF1A2 line B. Grip strength readings for forelimbs and all four limbs from three tests (measured in newtons) were normalized to body weight (measured in grams) and expressed × 100. *P < 0.01 for comparison of wasted mice without the transgene with wild‐type mice; those with the transgene are not significantly different from wild‐type mice. SEM, standard error of the mean.

Genotype at wasted locus	Transgene +/−	Grip strength ± SEM	No. of mice
Forelimbs only			
+/+	−	4.9 ± 0.2	13
+/+	+	4.4 ± 0.2	13
+/*wst*	−	4.5 ± 0.3	15
+/*wst*	+	4.9 ± 0.3	9
*wst*/*wst*	−	2.8 ± 0.3*	8
*wst*/*wst*	+	4.4 ± 0.4	6
All four limbs			
+/+	−	8.3 ± 0.4	13
+/+	+	8.3 ± 0.4	13
+/*wst*	−	7.5 ± 0.3	15
+/*wst*	+	8.4 ± 0.3	9
*wst*/*wst*	−	6.1 ± 0.4*	8
*st*/*wst*	+	8.6 ± 0.3*	6

By all measures applied, therefore, the NSE–eEF1A2 transgene was sufficient to correct the defects seen in wasted mice. However, even though expression of the transgene was enriched in brain as compared with muscle, it was still expressed at normal levels in muscle, so this experiment alone was not sufficient to establish whether the primary cause of disease in wasted mice is neurological in origin. It is worth noting that the lack of specificity of the NSE promoter that we observed is unexpected, but not unprecedented in the literature 22. We therefore sought to express eEF1A2 specifically in muscle to establish whether this too would result in correction of the wasted phenotype.

### HSA–eEF1A2

Pronuclear injection of the HSA–eEF1A2 construct into fertilized mouse oocytes gave rise to three independently derived founder mice that all transmitted the transgene. None of the three lines corrected any aspect of the wasted phenotype, in spite of expressing the transgene at levels comparable to those seen in wild‐type controls. One line was selected for further analysis.

**Figure 3:**
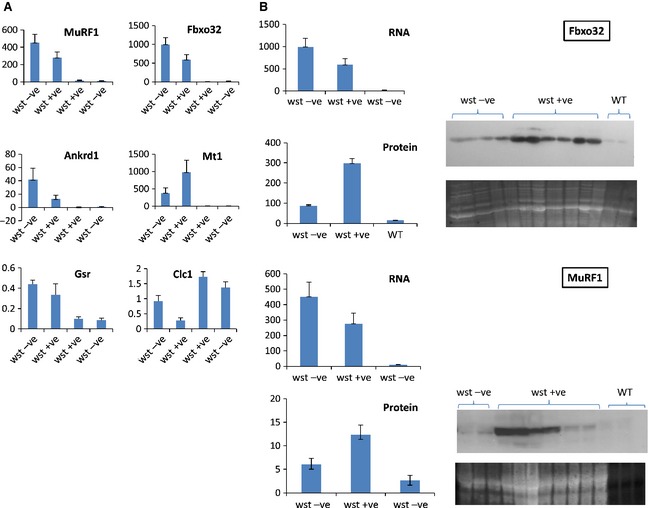
Western blots showing expression of eEF1A2 and GAPDH (as a loading control) in muscle and brain tissue extracts from transgenic HSA–EEF1A2 mice and nontransgenic mice. The first lane shows liver, as a negative control for eEF1A2 expression, and the third lane shows muscle or brain from a wasted nontransgenic mouse. The second lane shows the relevant tissue from a nontransgenic wild‐type mouse, followed by four wasted homozygotes carrying the transgene. The transgene is expressed at levels comparable to those seen in wild‐type mice in muscle, but no expression is seen in brain.

Western blot analysis of muscle and brain from transgenic *wst*/*wst* mice and controls shows that eEF1A2 was expressed in muscle of transgene‐positive wasted mice at levels comparable to those seen in control wild‐type mice (Fig. 3; densitometry gave an average ratio of 1.7 for wasted transgenic mice as compared with wild‐type mice). In contrast, analysis of brain extracts shows that, whereas endogenous eEF1A2 was easily detectable in wild‐type brain, no expression was seen in brain of transgenic wasted mice, suggesting that the HSA promoter operates in a muscle‐specific manner in these lines (note that the faint band seen in the brains of wasted transgenic mice in Fig. 3 is actually slightly smaller than eEF1A2, and the same band can also be seen in nontransgenic wasted control mice).

In spite of the presence of physiological levels of eEF1A2 in the muscle of transgenic *wst*/*wst* mice, no amelioration of the phenotype was seen. Growth curve analysis of all four groups of mice, wild‐type mice with and without the transgene, and *wst*/*wst* mice with and without the transgene, showed that wild‐type mice continued to put on weight until ~ 29 days (the end of the period studied), regardless of the presence or absence of the transgene. Wasted mice, in contrast, failed to gain weight after 22 days, and again, there was no effect of the transgene (Table 3), suggesting that there was no difference between the groups in terms of nutritional status. No wasted mice, transgenic or nontransgenic, survived beyond 29 days.

**Table 3:** Body weights of mice of different genotypes from an HSA–eEF1A2 line at 25 and 28 days. SEM, standard error of the mean.

Genotype at wasted locus	Days	Transgene +/−	Weight (g) ± SEM	No. of mice
+/+	25	−	11.7 ± 0.2	3
+/+	25	+	11.2 ± 0.9	5
*wst*/*wst*	25	−	6.8 ± 0.4	3
*wst*/*wst*	25	+	7.2 ± 0.5	3
+/+	28	−	12.7 ± 0.8	6
+/+	28	+	13.7 ± 0.4	5
*wst*/*wst*	28	−	7.7 ± 0.6	5
*wst*/*wst*	28	+	6.6 ± 0.8	3

Measurements of grip strength in these mice showed that the presence of physiological levels of eEF1A2 in muscle only had no measurable effect on muscle function. Wasted mice with or without the transgene performed significantly less well than wild‐type controls by ~ 27 days, with the rate of decline in forelimb strength in wasted transgenic mice exactly tracking that in nontransgenic wasted mice (Table 4; Fig. S2).

**Table 4:** Mean grip strength analysis of 25‐ and 28‐day‐old mice from an HSA–EEF1A2 line. Grip strength readings for forelimbs and all four limbs from three tests (measured in newtons) were normalized to body weight (measured in grams) and expressed × 100. *P < 0.01 for comparison with wild‐type nontransgenic mice. **P < 0.001 for comparison with wild‐type nontransgenic mice. SEM, standard error of the mean.

Genotype at wasted locus	Days	Transgene +/−	Grip strength ± SEM	No. of mice
Forelimbs only				
+/+	25	–	4.4 ± 0.8	4
+/+	25	+	4.6 ± 0.4	3
*wst*/*wst*	25	−	4.4 ± 0.4	3
*wst*/*wst*	25	+	3.3 ± 0.6	5
+/+	28	−	5.0 ± 0.3	5
+/+	28	+	4.4 ± 0.1	5
*wst*/*wst*	28	−	2.6 ± 0.5*	5
*wst*/*wst*	28	+	2.3 ± 0.5*	3
All four limbs				
+/+	25	−	8.0 ± 1.0	4
+/+	25	+	10.5 ± 0.7	3
*wst*/*wst*	25	−	6.1 ± 0.3	5
*wst*/*wst*	25	+	5.6 ± 0.5	10
+/+	28	−	9.4 ± 0.4	5
+/+	28	+	9.0 ± 0.2	5
*wst*/*wst*	28	−	5.0 ± 0.5**	5
*wst*/*wst*	28	+	4.8 ± 0.2**	3

**Figure 4:**
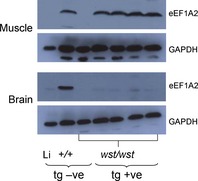
Periodic acid–Schiff‐stained muscle sections showing reduced fibre size in wasted mice (right), both with (bottom) and without (top) the muscle‐specific eEF1A2 transgene.

We then carried out an analysis of tissues and tissue sections from limb muscle of the same groups of mice at different ages. Wasted mice have previously been found to have reduced hind leg muscle mass relative to wild‐type mice from 23 days 7. Histologically, this correlated with a diffuse, nonregional reduction in myofibre cross‐sectional area (atrophy; Fig. 4). Expression of the transgene in *wst*/*wst* mice had no obvious effect on hind leg muscle mass at 25–26 days or at 26–28 days. The changes seen were consistent with neurogenic and disuse atrophy, but were too rapid and acute to impart specificity to the atrophy, given the shortness of the interval between onset of abnormality and death in wasted mice. No differences were observed between wild‐type mice with and without the transgene.

### Muscle atrophy and atrogene induction

Although there was no overt difference between wasted mice without the transgene and those in which eEF1A2 expression had been restored in muscle with the HSA promoter, we wanted to determine whether known biomarkers of muscle atrophy, such as those that have been shown to be associated with denervation atrophy in other mouse models of MND, showed any changes. We assayed these both in wasted mice as compared with their wild‐type littermates, and also in wasted mice with and without the transgene. A selection of markers was chosen, based on the literature. Atrogin 1 (*Fbxo32*) 16, muscle RING finger‐1 (*MuRF1*) 16, metallothionein 1 (*Mt1*) 24, metallothionein 2 (*Mt2*) 24 and glutathione reductase (*Gsr*) 25 have all been shown to be upregulated in conditions of muscle atrophy, whereas muscular chloride channel 1 (*Clc1*) has been shown to be downregulated in muscle disorders, including SMA 26.

**Figure 5:**
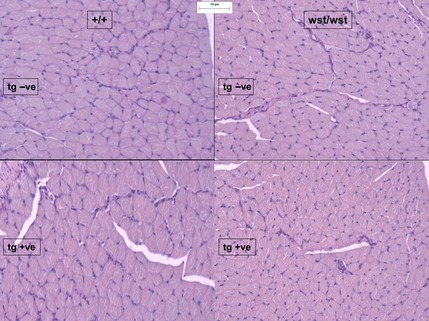
(A) qRT‐PCR data for markers of muscle atrophy in groups of mice of each genotype plus and minus the muscle‐specific eEF1A2 transgene. The *y*‐axis shows the expression level of the gene relative to the reference genes (arbitrary units). Six mice were analysed for both groups of wasted animals, and four or five for each wild‐type group. (B) Quantification of Fbxo32 (upper panel) and MuRF1 (lower panel) mRNA and protein levels in wasted mice with and without muscle‐specific expression of eEF1A2; western blot showing Fbxo32 as compared with Sypro Ruby loading control (arbitrary units).

The results obtained are shown in Fig. 5A. No marker showed any significant difference when wild‐type mice with and without the transgene were compared, as expected. There was high variability within each of the wasted mouse groups, and although there appeared to be a trend for markers to be more highly expressed in wasted mice without the transgene, there were no statistically significant differences between these groups. *Clc1* expression, however, which is downregulated in response to muscle atrophy, was significantly lower in wasted mice with the transgene than in those without (*P* = 0.03). This is counterintuitive, and suggests that the presence of the transgene is in some way deleterious.

In contrast, it can be seen that, for each of the markers shown, there was a highly significant change in expression in wasted mice as compared with their normal littermate controls. In the most extreme case, *Fbxo32*, there was 45‐ and 76‐fold upregulation of the gene, respectively, in wasted mice with the transgene (*P* = 0.009) and without the transgene (*P* = 0.005) as compared with wild‐type controls, which is consistent with a substantial degree of denervation‐induced atrophy in each group.

As eEF1A2, the factor deficient in wasted mice, has been shown *in vitro* to be a translation elongation factor 27, we next investigated whether these highly significant changes in gene expression seen at the RNA level would be reflected at the protein level. As muscle ceases to express eEF1A1 by approximately 3–4 weeks of age in mice, the prediction would be that wasted mouse muscle would cease to be capable of *de novo* protein synthesis at about this time, but this has never been directly tested. We chose *Fbxo32* to study, as the dramatic upregulation seen by qRT‐PCR made this a good candidate for detecting any differences between wasted mice without the transgene (and thus with a potentially diminished capacity for translation in muscle) and those with the transgene, which should have a normal capacity for protein synthesis. We also examined *MuRF1* expression. Both groups of mice were, of course, undergoing muscle atrophy to the same extent, as measured in the assays described above, and thus represent parallel systems showing muscle response to neuronal damage, but in the presence and absence of muscle eEF1A2.

Figure 5(B) shows the results obtained for *Fbxo32* and *MuRF1* at both the RNA level and the protein level. The western blot showed barely detectable protein levels in wild‐type mice, and substantially more Fbxo32 in wasted mice. However, the increase was less than sixfold (*P* = 0.0009), in contrast to the 76‐fold upregulation seen at the RNA level. This was shown more dramatically in the comparison with wasted mice with eEF1A2 expression in muscle; here, Fbxo32 protein levels were, in fact, upregulated 20‐fold as compared with wild‐type controls (*P* = 0.0006), a value comparable to the 45‐fold upregulation seen at the RNA level. There was a highly significant difference in the Fbxo32 protein levels seen in wasted mice with and without the transgene (*P* = 0.0001). Similar results were observed with MuRF1. Here, there was 40‐fold upregulation in nontransgenic wasted mice at the RNA level (*P* = 0.009) but nonsignificant upregulation at the protein level (*P* = 0.13); transgenic wasted mice, on the other hand, showed 24‐fold upregulation at the RNA level but nearly fivefold upregulation at the protein level (*P* = 0.016; Fig. 5B).

Thus, although there is certainly some conversion of the RNA‐level upregulation seen in wasted mice into protein, this occurs to a much lesser extent in muscles that have no eEF1A2, and this represents the first *in vivo* evidence of the physiological role of eEF1A2 in translation. Furthermore, the relatively low levels of Fbxo32 and MuRF1 proteins, as opposed to their mRNAs, in wasted muscle suggest that the process of muscle atrophy can occur without significant upregulation of at least this key atrogene at the protein level.

## Discussion

Our study shows that loss of eEF1A2 leads to muscle atrophy resulting from loss of eEF1A2 in neurons. We have demonstrated that, whereas expression of an eEF1A2 transgene in neuronal and muscle tissue completely corrects the wasted phenotype in terms of muscle function and survival, physiological levels of expression of eEF1A2 in skeletal muscle have no effect on the course of disease progression or time of death. Whereas mice with muscle‐only expression of eEF1A2 die by 4 weeks, those with both neuronal and muscle expression are able to survive with no apparent phenotypic abnormalities for 12 months. As the same results were obtained in lines generated from three independent founders, we can have some confidence in these conclusions, even in the absence of a neuron‐specific correction.

Grip strength measurement was a useful and sensitive assay for muscle function in this study. Wasted mice showed a clear and consistent loss of grip strength in comparison with their wild‐type littermates. In the NSE line experiments, which were conducted a year before the HSA experiments, the difference was clear by 25 days, with no overlap between the groups. In the HSA experiments, the wasted mice generally survived for a day or two longer, so the difference between genotypes was less clear at 25 days, but very marked by 27 days. This fitted well with the pathological observations of widespread atrophy in the muscles of wasted mice, which remained unaffected by the presence of high levels of eEF1A2 transgene expression in muscle.

Our results are similar to a previous study of SMN, although here the HSA promoter construct gave rise to high levels of expression in muscle but leaky expression in brain and spinal cord in certain lines, and the neuronal promoter used, PrP, also gave leaky expression in muscle. This study also concluded that expression of high levels of SMN in muscle fibres alone had no effect on the SMA phenotype or survival of the mice in which the correction experiments were performed 8.

In contrast, studies of a spontaneously arising model of SMA with respiratory distress, the neuromuscular degeneration (*nmd*) mutant mouse, showed that, whereas transgenic expression of *Ighmbp2* in neurons was sufficient to correct the motor neuron degeneration in the mice, they subsequently developed skeletal myopathy and dilated cardiomyopathy. When a muscle‐specific transgene was introduced, this extended the lifespan of the mice eightfold. However, correction of the full phenotype, including motor neuron degeneration, required the expression of *Ighmbp2* transgenes in both neuronal and muscle tissue 9. These experiments suggest that the early lethality of *nmd*/*nmd* mice is primarily attributable to the development of dilated cardiomyopathy rather than the progressive MND that is also seen.

Our studies of wasted mice, on the other hand, suggest that the phenotypic abnormalities, including the muscle pathology and muscle function, result from the loss of eEF1A2 expression in neurons. This makes wasted mice a useful addition to the spectrum of animal models of motor neuron degeneration, particularly in view of the early onset and aggressive nature of the phenotype. It should also be borne in mind, however, that the presence of eEF1A2 in muscle could make some contribution to the phenotype, and that correction of expression in neurons would not fully ameliorate the phenotypic abnormalities.

Analysis of expression levels of genes involved in muscle atrophy induced by motor neuron degeneration, surgical denervation and other triggers provides further insights into the function of eEF1A2, and into the mechanisms of muscle atrophy. First, the 76‐fold upregulation of *Fbxo32* seen in muscle of wasted mice as compared with wild‐type littermates results in a less than sixfold upregulation at the protein level. This contrasts with the situation seen in wasted mice with restored expression of eEF1A2 in muscle, which show a smaller, 45‐fold change in *Fbxo32* mRNA, and yet a 20‐fold change in the level of Fbxo32 protein. Thus, whereas *Fbxo32* mRNA levels are almost twice as high in wasted mice without the transgene, Fbxo32 protein levels are over threefold lower in these mice than in those expressing eEF1A2 in muscle. This strongly suggests that the absence of eEF1A2 in muscle severely compromises the ability of this tissue to carry out *de novo* protein synthesis, confirming, for the first time *in vivo*, the biochemical characterization of eEF1A2 as a translation elongation factor. The modest induction that is seen in wasted muscle is presumably attributable to residual amounts of eEF1A1; although this has largely gone by 21 days, retraction of nerves from motor nerve endplates is seen from at least 17 days, so the trigger for upregulation of *Fbxo32* would precede the complete downregulation of eEF1A1.

Importantly, we can use this system of wasted mice with (HSA–eEF1A2 transgene) and without (nontransgenic) eEF1A2 in muscle as a system for studying the molecular basis of the programme of events underlying muscle atrophy. Deletion of either *Fbxo32* or *MuRF1* in mice ameliorates the muscle damage resulting from denervation over a period of 7–14 days, suggesting that the products of these genes are necessary for the execution of the full programme of muscle atrophy 18. The genes encode ubiquitin ligases, whose role is to target contractile proteins for degradation by the proteasome. Whereas approximately similar, extremely high, levels of induction are seen at the mRNA level in wasted mice with and without an eEF1A2 transgene in muscle, there is a 3.4‐fold difference in the level of Fbxo32 protein seen in these two groups of mice. The degree of upregulation in the nontransgenic mice is very modest, especially when compared with the qRT‐PCR results, and yet atrophy as determined by pathology and grip strength is indistinguishable between the transgenic and nontransgenic wasted mice. As there is no reason to think that the mRNAs for these genes are functional, these data suggest that, although these atrogenes may be essential for many forms of muscle atrophy, this is not inevitably the case. We suggest that, perhaps, loss of protein synthetic capacity in muscle should be considered as passive muscle atrophy, whereas mechanisms involving atrogene induction and proteolysis should be considered to be active muscle atrophy. Further studies using this system are likely to be very useful for understanding atrogene function.

The question of whether wasted mice constitute a useful model for motor neuron degeneration remains an open one, although our results certainly point more to a neurodegenerative phenotype than to one of primary muscle atrophy. A common (and valid) criticism of the model is that no mutations have been found in the gene encoding eEF1A2 in human ALS patients. However, the recent discoveries 11 of a heterozygous missense mutation in eEF1A2 in two separate individuals with intractable early‐onset epilepsy and severe intellectual disabilities may shed some light on this. The mutation is predicted to give rise to loss of function; if this is the case, then homozygous loss of function mutations (equivalent to wasted mice) would be unlikely to be seen in human populations.

## Experimental procedures

### Constructs

A pUC19–EEF1A2 plasmid was created by subcloning the 18 891‐bp *eEF1A2* gene from a human PAC. This backbone was used for generation of the transgenic construct. An *Age*I site located 102 bp upstream of the second exon (the first coding exon) of eEF1A2 was identified as a suitable unique site for insertion of the new promoter. The promoter region of *eEF1A2* was removed by digestion with *Hin*dIII and *Age*I. The NSE promoter (a 1946‐bp fragment of the rat NSE promoter generated by PCR and flanked with *Hin*dIII and *Age*I sites) was then inserted into this site. The HSA promoter was a kind gift from J. Tinsley (MRC Harwell, Oxfordshire, UK) 28, and covered the region −2000 to +239. The transgene constructs were excised from the vector and gel purified, diluted to 2 ng·μL^−1^ with microinjection buffer, and purified in a Spinex column in preparation for microinjection.

### Mice

Mice were housed in the Biomedical Research Facility at the University of Edinburgh. All mice were maintained in accordance with Home Office regulations, and all protocols were approved by the local ethics committee of the University of Edinburgh. Embryo transfer was carried out with short‐term recovery anaesthesia, and analgesia when needed postoperatively. Transgenic and mutant mice were closely observed for overall clinical condition, and were killed when necessary to avoid suffering.

### Transgenic mouse generation

Transgenic mice were made by standard pronuclear injection into oocytes derived from (C57BL/6 × CBA)F_1_ mice. Founder mice that carried an intact transgene as established by PCR genotyping and expression analysis were then crossed to +/*wst* mice, and then intercrossed to derive transgene‐positive *wst*/*wst* mice.

### Grip strength

A grip strength meter (Bioseb, http://www.bioseb.com) was used to measure limb muscle strength. Forelimbs and all four limbs were measured separately by lowering the mouse onto the grid until it grasped it with either front limbs or all limbs. The tail was then pulled steadily from the grid in one fluid movement until the mouse released its grip. The maximum force in newtons required for the mouse to relinquish its grip was recorded, and the readings were normalized to body weight. Measurements were made three times per mouse, with a 1‐min break between trials, and the results were averaged. Significance was estimated by *t*‐tests with prism graphpad (San Diego, CA, USA).

### Western blots

Western blotting was carried out with antibodies and techniques as previously described 6, with the addition of anti‐eEF1A2 Ig from Genetex (Irvine, CA, USA) (1 : 2000) and anti‐Fbxo32 Ig from Abcam (Cambridge, UK) (1 : 2000). Calbiochem (Darmstadt, Germany) Rapid Step ECL was used for detection, and either GAPDH (Chemicon, Watford, UK; 1 : 30000) or Sypro Ruby (later blots) were used as loading controls. Quantification of band intensity was carried out with genesnap (Cambridge, UK) and genetool (Cambridge, UK), or image j (http://rsbweb.nih.gov/ij/), and significance assessed with two‐tailed *t*‐tests for samples of equal variance.

### Pathology

Sections of deep digital flexor forelimb muscle were prepared from wasted, transgenic and control mice, and stained with Periodic acid–Schiff.

### qRT‐PCR

RNA was extracted from mouse quadriceps muscle with the RNeasy Mini Kit (Qiagen, Manchester, UK). During extraction, RNA was treated for 15 min with DNase I (Qiagen) to minimize any DNA contamination. The RNA concentration in samples was assessed with a NanoDrop 1000 device (Thermo Scientific, Loughborough, UK). In order to synthesize cDNA from mRNA, the High‐Capacity cDNA Reverse Transcription Kit (Applied Biosystems, Paisley, UK) was used, according to the manufacturer's instructions. qRT‐PCR was performed with a standard curve method for the quantification of gene expression. Primer sequences were taken from the literature or commercial sources as follows: *Fbxo32* 29, *Ankrd1* 30, *Gsr* 31, *Mt1* 32, *Cic1* 33, *Tubb* 34, and *MuRF1* (Qiagen assay QT00291991) All qRT‐PCR assays complied with the Minimum Information for Publication of Quantitative Real‐Time PCR experiments guidelines 35. For gene expression normalization, we used the geometric average of the combination of the two most stable reference genes, β_2_‐microglobulin (*B2 m*), and β‐tubulin (*Tubb2*). These genes were identified with the genorm program 36. PCR product accumulation was detected with fluorescent SYBR Green dye (Finnzymes, Loughborough, UK). A series of diluted cDNAs was used to construct a standard curve for each pair of primers, and only those with efficiencies from 90% to 105% were used for further analysis. The result for each target gene was normalized against the reference genes, and the ΔΔC_T_ method was used to determine relative changes in transcriptional expression. Significance testing was carried out with the Mann–Whitney/Wilcoxon rank sum test for populations that are not normally distributed.

## Supplementary Material

**Fig. S1.** Immunohistochemistry of eEF1A2 in sections from spinal cord, brain and pancreas from mice of different genotypes.
**Fig. S2.** Grip strength values for wasted HSA‐eEF1A2 transgenic and nontransgenic mice, and wild‐type transgenic and nontransgenic controls, measured from weaning until 30 days.
