# Supplementary material for: In vivo characterization of the role of tissue‐specific translation elongation factor 1A2 in protein synthesis reveals insights into muscle atrophy
Source: FEBS J. 2013 Oct 23;280(24):6528–40. doi: 10.1111/febs.12554 (PMC4163635; doi:10.1111/febs.12554)

the FEBS Journal
www.febsjournal.org

In vivo characterization of the role of tissue-specific translation elongation factor 1A2 in protein synthesis reveals insights into muscle atrophy

Jennifer Doig, Lowri A. Griffiths, David Peberdy, Permphan Dharmasaroja, Maria Vera2, Faith J. C. Davies, Helen J. Newbery, David Brownstein and Catherine M. Abbott

DOI: 10.1111/febs.12554

Figure S1

Immunohistochemistry of eEF1A2 in sections from spinal cord, brain and pancreas from mice of different genotypes. Sections from wild type mice are shown in the top panel, wasted mice (showing no eEF1A2 expression) in the middle panel, and wasted mice expressing an eEF1A2 transgene in muscle and brain in the bottom panel. Note that the transgene, unlike the endogenous eEF1A2 gene, is not expressed in pancreatic islet cells.

Figure S2

Grip strength values for wasted HSA-eEF1A2 transgenic and non-transgenic mice, and wild-type transgenic and non-transgenic controls measured from weaning until 30 days. Upper panel shows values for forelimbs only and lower panel shows values for all four limbs.

Fig S1

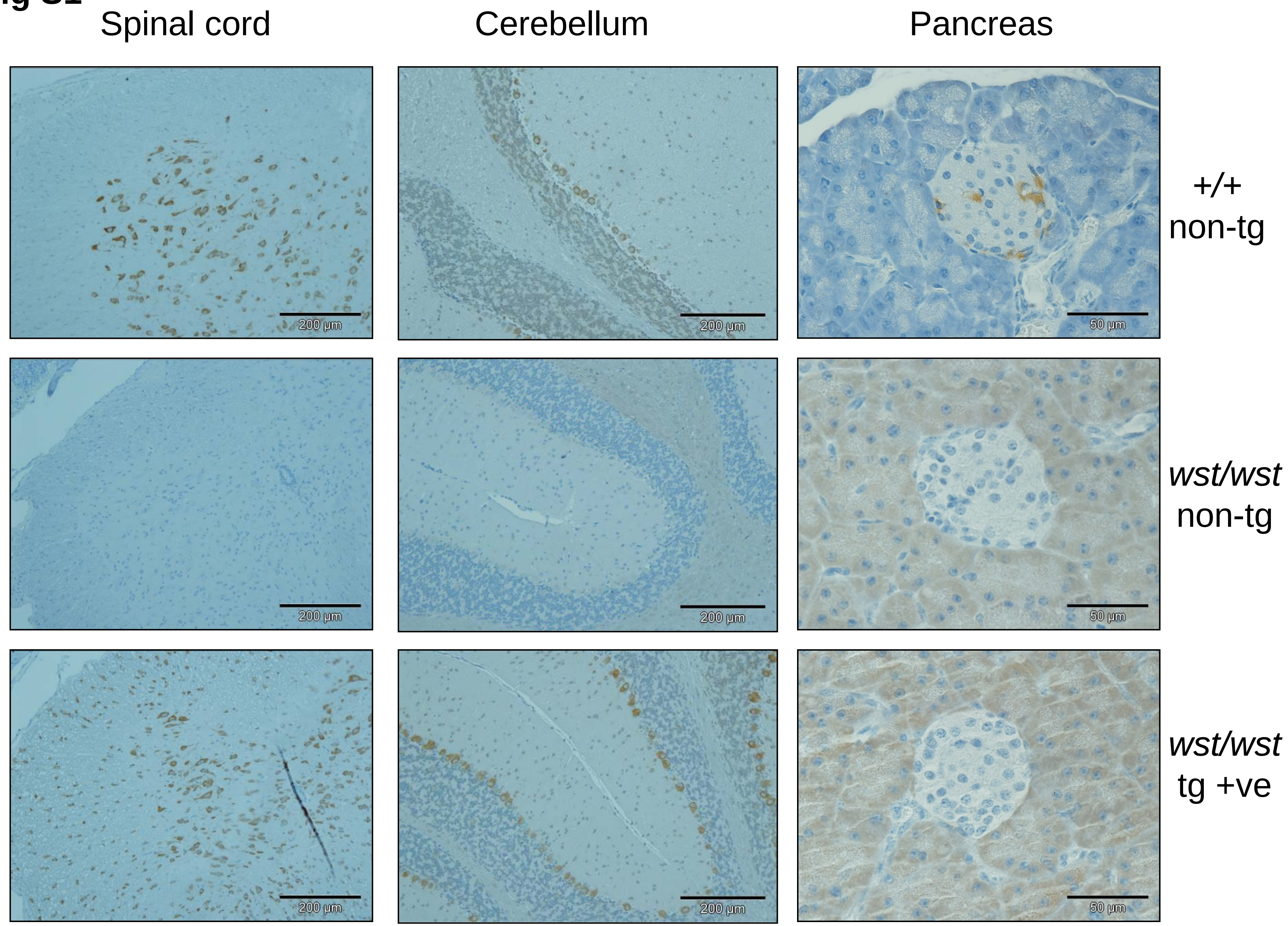
Spinal cord
Cerebellum
Pancreas
+/+
non-tg
wst/wst
non-tg
wst/wst
tg +ve
200 µm
50 µm

Grip Strength in fore Limbs

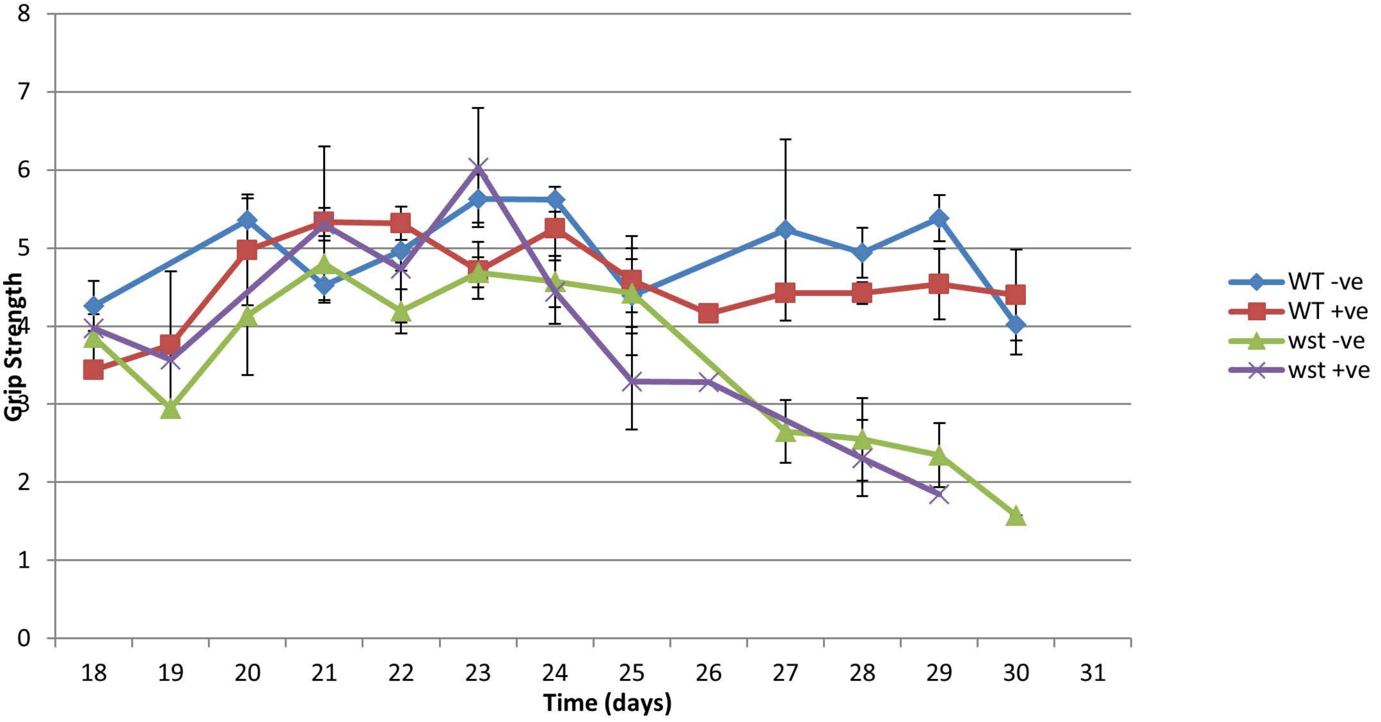

Grip Strength in all four limbs

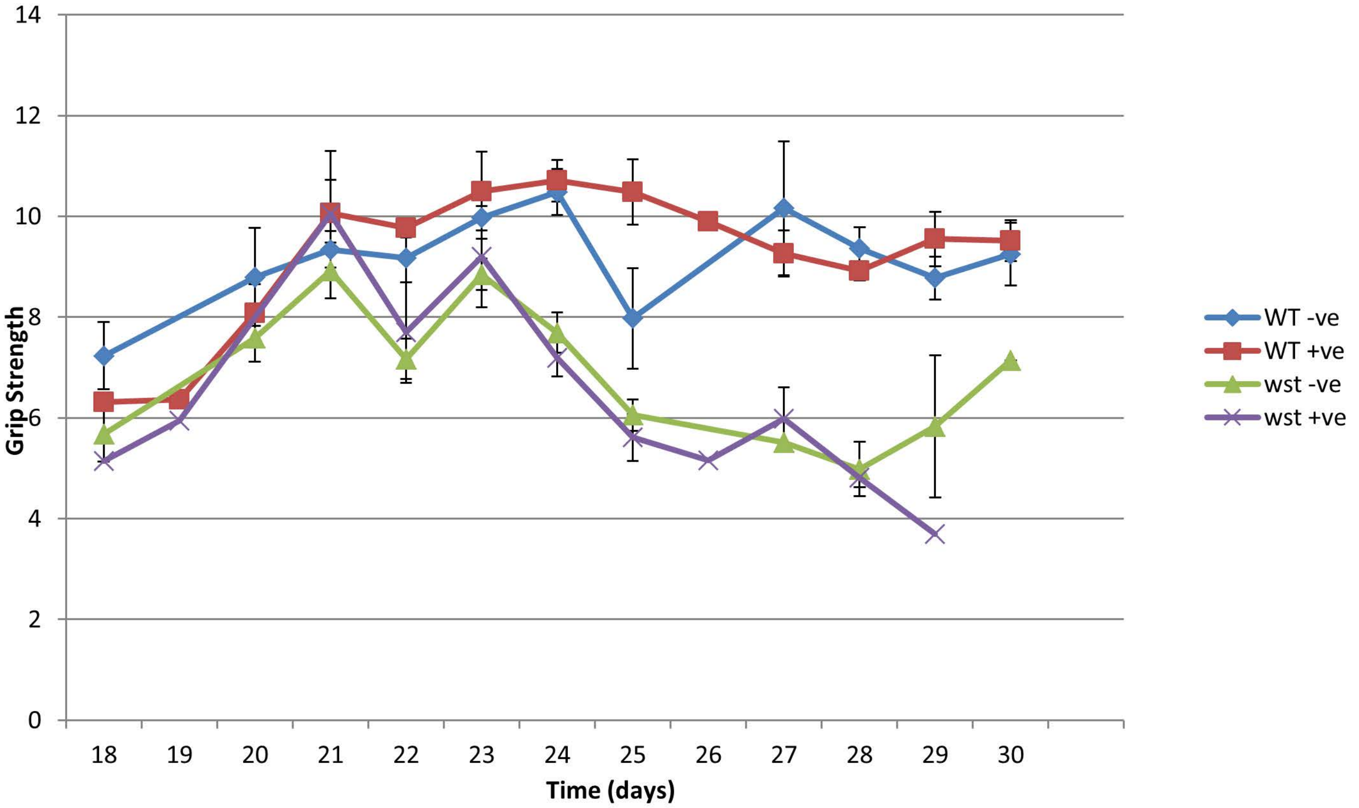

Supplement: Supplementary file 1 — Fig. S1. Immunohistochemistry of eEF1A2 in sections from spinal cord, brain and pancreas from mice of different genotypes. Fig. S2. Grip strength values for wasted HSA‐eEF1A2 transgenic and nontransgenic mice, and wild‐type transgenic and nontransgenic controls, measured from weaning until 30 days. [file febs-280-6528-s1.zip › febs12554-sup-0001-FigS1-S2.pdf]
